# Genome-Wide Identification and Expression Analysis of ACTIN Family Genes in the Sweet Potato and Its Two Diploid Relatives

**DOI:** 10.3390/ijms241310930

**Published:** 2023-06-30

**Authors:** Shuanghong Xia, Huan Zhang, Shaozhen He

**Affiliations:** 1 Key Laboratory of Sweet Potato Biology and Biotechnology, Ministry of Agriculture and Rural Affairs/Beijing Key Laboratory of Crop Genetic Improvement/Laboratory of Crop Heterosis & Utilization and Joint Laboratory for International Cooperation in Crop Molecular Breeding, Ministry of Education, College of Agronomy & Biotechnology, China Agricultural University, Beijing 100193, China; m18518713006@163.com (S.X.); zhanghuan1111@cau.edu.cn (H.Z.); 2 Sanya Institute of China Agricultural University, Sanya 572025, China

**Keywords:** sweet potato, *I. trifida*, *I. triloba*, *ACTIN*, tissue-specific expression, tuberous root development, hormone treatment, abiotic stress

## Abstract

ACTINs are structural proteins widely distributed in plants. They are the main components of microfilaments and participate in many crucial physiological activities, including the maintenance of cell shape and cytoplasmic streaming. Meanwhile, *ACTIN*, as a housekeeping gene, is widely used in qRT-PCR analyses of plants. However, *ACTIN* family genes have not been explored in the sweet potato. In this study, we identified 30, 39, and 44 *ACTINs* in the cultivated hexaploid sweet potato (*Ipomoea batatas*, 2n = 6x = 90) and its two diploid relatives, *Ipomoea trifida* (2n = 2x = 30) and *Ipomoea triloba* (2n = 2x = 30), respectively, via analysis of their genome structure and by phylogenetic characterization. These ACTINs were divided into six subgroups according to their phylogenetic relationships with *Arabidopsis thaliana*. The physiological properties of the protein, chromosome localization, phylogenetic relationship, gene structure, promoter *cis*-elements, protein interaction networks, and expression patterns of these 113 *ACTINs* were systematically investigated. The results suggested that homologous *ACTINs* are differentiated in the sweet potato and its two diploid relatives, and play various vital roles in plant growth, tuberous root development, hormone crosstalk, and abiotic stress responses. Some stable *ACTINs* that could be used as internal reference genes were found in the sweet potato and its two diploid relatives, e.g., *IbACTIN18*, *-20*, and *-16.2*; *ItfACTIN2.2*, *-16*, and *-10*; *ItbACTIN18* and *-19.1*. This work provides a comprehensive comparison and furthers our understanding of the *ACTIN* genes in the sweet potato and its two diploid relatives, thereby supplying a theoretical foundation for their functional study and further facilitating the molecular breeding of sweet potatoes.

## 1. Introduction

ACTIN is an ancient and highly conserved protein present in all eukaryotic cells. Moreover, ACTIN is a major component of the cytoskeleton and is the basis for the appearance of cells, tissue, and normal growth [1,2,3]. ACTIN also displays an irreplaceable role in connecting functional proteomic and life processes of cell [4,5,6]. In addition, ACTIN is a highly conserved protein of approximately 42 kDa, and its polypeptide chain has a molecular weight of 375 amino acids. It has two biological states: globular monomer G-ACTIN and filamentous F-ACTIN. G-ACTIN is the free globular monomer of ACTIN that is soluble and can be polymerized to form F-ACTIN. The F-ACTIN filament forms both the cytoskeleton and the contractile apparatus of muscle cells and is responsible for cell mobility and muscular contractions. An isoelectric focusing electrophoresis analysis showed that ACTIN has three main isotypes (α-ACTIN, β-ACTIN, and γ-ACTIN). ACTINs are characterized by abundant acidic amino acids with an isoelectric point of about 5.5 [7]. The 375-amino-acid (aa) polypeptide chain of ACTIN, which is common to Hsp70, MreB, ParM, and Arp proteins, folds into two major α/β-domains. ADP or ATP is located in the cleft between the domains, with a calcium ion bound to the β-, or β- and γ-phosphates, respectively [8]. However, plant ACTINs are encoded by multiple genes, resulting in a variety of ACTIN isoforms [9,10]. The isoforms of ACTIN differ by only a few amino acids, with most variations occurring toward the N-terminus [11]. ACTIN also undergoes various post-translational modifications. For instance, the N-terminal methionine and cysteine residues are acetylated and cleaved, and the resulting N-terminal aspartic acid is then reacetylated [7].

ACTINs, crucial structural proteins, play vital roles in various cellular functions such as cell division, cell movement, endocytosis, nucleation, cell signal transduction, gravity induction, and diverse cell movements including apical growth in organisms and organelle movement [12,13,14]. The *Arabidopsis thaliana*, rice (*Oryza sativa*), poplar (*Populus trichocarpa*), and grape (*Vitis vinifera*) genomes encode 20, 22, 18, and 16 ACTINs, respectively [15,16]. They are divided into six subfamilies in the *Arabidopsis thaliana*, five of which exhibit tissue-specific expression patterns [17]. For instance, *ACT1* and *ACT3* are expressed in mature pollen and all organ primordia, *ACT4* and *ACT12* are expressed in mature pollen and young vascular tissue, and *ACT11* is highly expressed in ovule, embryo, and endosperm [18]. *ACT2* and *ACT8* are expressed in most vegetative tissues [17,19,20]. Interestingly, the ectopic expression of *ACT1* resulted in plant dwarfing and altered organ morphology [21]. Double mutants of vegetative *ACT2* and *ACT8* (*act2/8*) in *Arabidopsis thaliana* exhibited an increase in the leaf area and ploidy level of mature leaves [22]. Knock-down of *ACT2* and *ACT8* resulted in totally hairless roots [23]. ACTIN acts as a fundamental component of the organelle skeleton and is essential for the vital processes of plants. It is often employed as an internal reference gene in plants due to its stable expression in various physiological states of the cell [24,25,26,27,28]. However, the biological functions and regulatory mechanisms of ACTINs remain unclear in the sweet potato.

The sweet potato (*Ipomoea batatas* (L.) Lam., 2n = B1B1B2B2B2B2 = 6x = 90), which belongs to the family Convolvulaceae, is an important food source for both humans and domesticated animals, as well as a new source of bioenergy in the form of bioethanol for fuel production [29]. It is planted in more than 100 countries or regions worldwide [30]. However, sweet potato is a hexaploid (2n = 6x = 90) with the characteristics of a complex genome, incompatibility with hybridization, a lack of germplasm resources, and susceptibility to diseases and insect pests. Therefore, the yield and quality of sweet potato have reduced due to various environmental pressures [31,32]. In recent years, the genomes of the hexaploid sweet potato and two diploid species, namely *I. trifida* NCNSP0306 (2n = 2x = 30) and *I. triloba* NCNSP0323 (2n = 2x = 30) [33], have been sequenced, assembled, and released, making it possible to identify and analyze important gene families at the whole-genome level in the sweet potato.

In this study, 113 *ACTINs* (30 in *I. batatas*, 39 in *I. trifida*, and 44 in *I. triloba*) were identified from the cultivated hexaploid sweet potato and its two diploid relatives. The physiological characteristics of the protein, chromosome location, phylogenetic relationships, conserved motifs, promoter *cis*-elements, and protein interaction networks of ACTINs in the sweet potato were systematically studied. In addition, analyses of the tissue specificity and expression patterns for the development of tuberous roots in different varieties of *ACTINs* were carried out. The results may play an important guiding role in the further study of their functions and the molecular breeding of sweet potatoes.

## 2. Results

### 2.1. Identification and Characteristic of ACTINs in the Sweet Potato and Its Two Diploid Relatives

To comprehensively identify all the ACTINs in the sweet potato and its two diploid relatives, we used three typical strategies (i.e., BLASTp searches, hmmer searches, and the CD-search database). In total, 113 *ACTINs* were identified, 30 in *I. batatas*, 39 in *I. trifida*, and 44 in *I. triloba*, which were named “*Ib*”, “*Itf*”, and “*Itb*”, respectively. Their physicochemical properties were analyzed using the sequence of *IbACTINs* (Table 1). The length of the CDS of *IbACTINs* varied from 345 bp (*IbACTIN5.4*) to 2196 bp (*IbACTIN10.2*). The lengths of the amino acids of IbACTINs ranged from 113 aa (IbACTIN5.4) to 720 aa (IbACTIN10.2), and the molecular weight (MW) ranged from 12.45 kDa (IbACTIN5.4) to 82.66 kDa (IbACTIN10.2). The isoelectric point (pI) varied from 4.71 (IbACTIN16.1) to 9.78 (IbACTIN7), but the pI of most IbACTINs was below 7, except for IbACTIN5.4, IbACTIN19, IbACTIN12.2, IbACTIN12.3, IbACTIN6, and IbACTIN7, suggesting that they are acidic proteins. All the IbACTINs contained Ser, Thr, and Tyr phosphorylation sites. Half of the IbACTINs were stable, with an instability index of less than 40, and the instability index varied from 31.71 (IbACTIN17.1) to 69.01 (IbACTIN7). The aliphatic index varied from 68.32 (IbACTIN10.1) to 106.02 (IbACTIN5.4); furthermore, the aliphatic index of most IbACTINs was more than 80, indicating that they are thermophilic proteins [34]. The grand average of hydropathicity (GRAVY) of all the IbACTIN proteins varied from −0.749 (IbACTIN10.2) to 0.15 (IbACTIN5.4), indicating that they are hydrophilic. A subcellular localization prediction assay showed that most of the IbACTINs were located in the cytoplasm and cytoskeleton but also in the chloroplast (IbACTIN6, -7, -12.2, -18, and -19) and nucleus (IbACTIN2.1, -2.2, and -12.3).

**Figure 1:**
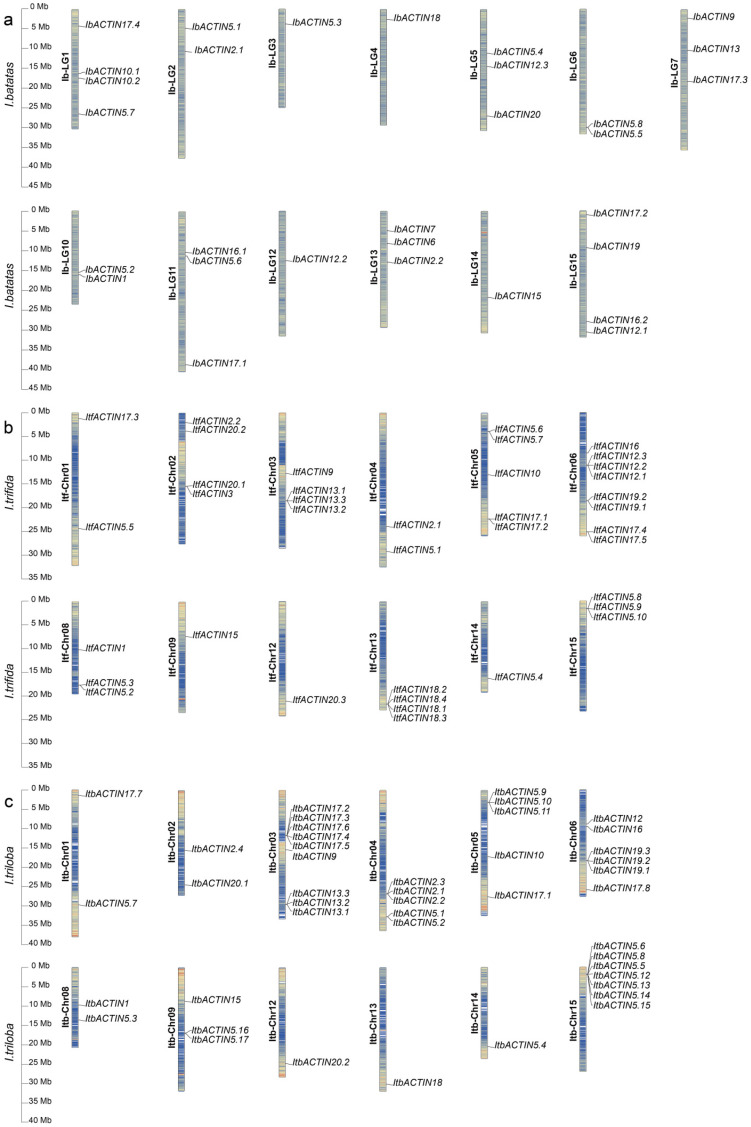
Chromosomal localization and distribution of *ACTINs* in *I. batatas* (**a**), *I. trifida* (**b**), and *I. triloba* (**c**). The bars represent chromosomes. The chromosome numbers are displayed on the left side, and the gene names are displayed on the right. Each gene location is shown on the line. Detailed chromosomal location information is listed in Table S1.

The *ACTINs* were distributed across 13, 12, and 12 chromosomes of *I. batatas*, *I. trifida*, and *I. triloba*, respectively (Figure 1). In *I. batatas*, four *IbACTINs* were detected on LG1 (*IbACTIN17.4*, *-10.1*, *-10.2*, and *-5.7*) and LG15 (*IbACTIN17.2*, *-19*, *-16.2*, and *-12.1*); three on LG5 (*IbACTIN5.4*, *-12.3*, and *-20*), LG7 (*IbACTIN9*, *-13*, and *-17.3*), LG11 (*IbACTIN16.1*, *-5.6*, and *-17.1*), and LG13 (*IbACTIN7*, *-6*, and *-2.2*); two on LG2 (*IbACTIN5.1* and *-2.1*), LG6 (*IbACTIN5.8* and *-5.5*), and LG10 (*IbACTIN5.2* and *-1*); and one on LG3 (*IbACTIN5.3*), LG4 (*IbACTIN18*), LG12 (*IbACTIN12.2*), and LG14 (*IbACTIN15*); whereas no genes were detected on LG8, and LG9 (Figure 1a). In *I. trifida* and *I. triloba*, the distribution of *ACTINs* on Chr01 (2) (*ItfACTIN17.3*, *-5.5* and *ItbACTIN17.7*, *-5.7*), Chr05 (5) (*ItfACTIN5.6*, *-5.7*, *-10*, *-17.1*, and *-17.2* and *ItbACTIN5.9*, *-5.10*, *-5.11*, *-10*, and *-17.1*), Chr12 (1) (*ItfACTIN20.3* and *ItbACTIN20.2*), and Chr14 (1) (*ItfACTIN5.4* and *ItbACTIN5.4*) was similar (Figure 1b,c). Notably, *itf00g14730* (*ItfACTIN20.4*) was not located on the regular 15 chromosomes. The results indicated that the variation in and a loss of *ACTINs* during evolution, caused the differences in the distribution and disproportion of *ACTINs* on the chromosomes between sweet potato and its two diploid relatives.

### 2.2. Phylogenetic Relationships of ACTINs in the Sweet Potato and Its Two Diploid Relatives

**Figure 2:**
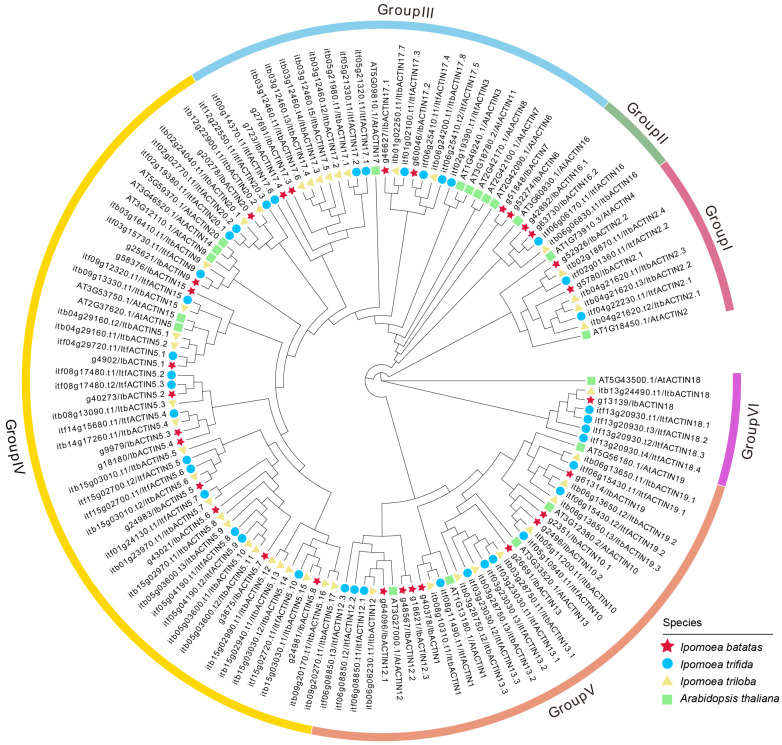
Phylogenetic analysis of the ACTIN proteins from four plant species (i.e., *I. batatas*, *I. trifida*, *I. triloba*, and *Arabidopsis thaliana*). In total, 133 ACTINs were divided into six subgroups (Group I to Group VI) according to the evolutionary distance. The crimson stars, blue circles, yellow triangles, and green rectangles represent the 30 IbACTINs in *I. batatas*, the 39 ItfACTINs in *I. trifida*, the 44 ItbACTINs in *I. triloba*, and the 20 AtACTINs in *Arabidopsis thaliana*, respectively.

To study the evolutionary relationship of the ACTINs in *I. batatas*, *I. trifida*, *I. triloba*, and *Arabidopsis thaliana*, we constructed a phylogenetic tree for 133 ACTINs of these four species (i.e., 30 in *I. batatas*, 39 in *I. trifida*, 44 in *I. triloba*, and 20 in *Arabidopsis thaliana*) (Figure 2). All the ACTINs were unevenly distributed on each branch of the phylogenetic tree, and they were divided into six subgroups (Groups I to VI), according to the evolutionary distance. The specific distribution of the ACTINs was as follows (total: *I. batatas*, *I. trifida*, *I. triloba*, and *Arabidopsis thaliana*): Group I (10: 2, 2, 4, 2), Group II (5: 2, 1, 1, 1), Group III (27: 6, 7, 8, 6), Group IV (52: 11, 15, 21, 5), Group V (32: 8, 10, 9, 5), and Group VI (7: 1, 4, 1, 1) (Figure 2; Table S1). We named the IbACTINs, ItfACTINs, and ItbACTINs on the basis of their homology with their homologs in *Arabidopsis thaliana*, and only AtACTIN1/2/3/5/6/7/9/10/12/13/15/16/17/18/19/20 from *Arabidopsis thaliana* had homologous proteins in *I. batatas*, *I. trifida*, and *I. triloba*. These results indicated that the number and type of ACTINs distributed in each subgroup of sweet potato differed from those of its two diploid relatives and *Arabidopsis thaliana*.

### 2.3. Analysis of Conserved Motifs and Exon-Intron Structure of ACTINs in the Sweet Potato and Its Two Diploid Relatives

**Figure 3:**
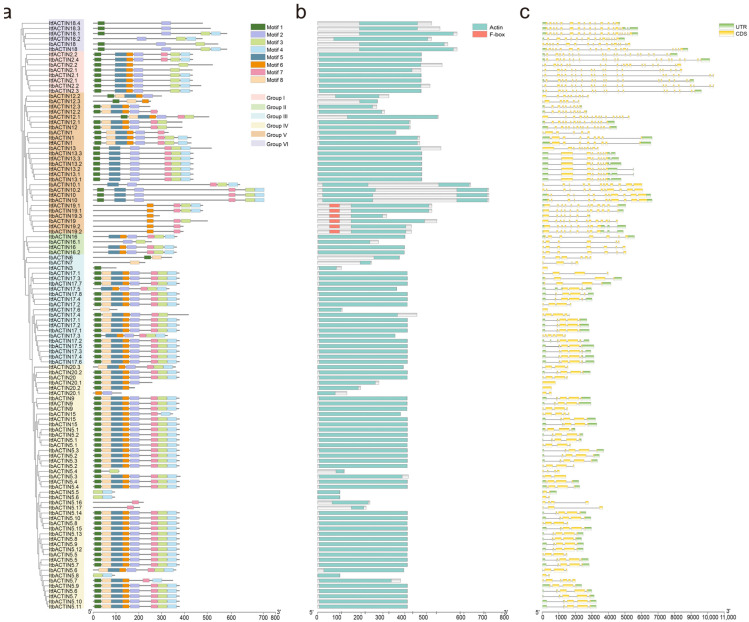
Conserved motifs and analysis of the exon-intron structure of the ACTIN family in *I. batatas*, *I. trifida*, and *I. triloba.* (**a**) The phylogenetic tree shows that the ACTINs are distributed in six subgroups (left), and the eight conserved motifs are shown in different colors. (**b**) Conserved domain structures of ACTINs. The blue-green box represents the ACTIN domain. The pink box represents the F-box domain. (**c**) Exon-intron structures of *ACTINs*. The green boxes, yellow boxes, and black lines represent the UTRs, exons, and introns, respectively.

Furthermore, sequence motifs in the 30 IbACTINs, 39 ItfACTINs, and 44 ItbACTINs were analyzed using the MEME website, and the eight most conserved motifs were identified (Figure 3a and Figure S1). Most of the ACTINs (50 ACTINs) contained these eight conserved motifs. We found that ACTINs in the same subgroup have similar conserved motifs, whereas there were differences in the types of motifs between each subgroup.

The ACTINs in Group I had Motif 1, Motif 2, Motif 3, Motif 4, Motif 5, and Motif 6, except for IbACTIN2.2 (which lacked Motif 2 and Motif 4), IbACTIN2.1 (which lacked Motif 4), ItbACTIN2.4 (which contained Motif 7), and ItfACTIN2.2 (which contained Motif 7). In Group II, IbACTIN16.1 contained Motif 2 and Motif 3, while the other ACTINs contained Motif 2, -3, -4, -5, -6, and -7. Most of the ACTINs in Group III and Group IV had eight motifs, and the members of the subgroup were relatively conservative. In Group III, only seven ACTINs lacked at least one motif; the other ACTINs had eight motifs. In Group IV, most ACTINs had 8 motifs, except for 13 ACTINs (e.g., IbACTIN5.4 contained Motif 1 and Motif 3). The motif positions of IbACTIN12.1, -12.2, and -12.3 were different from those of other proteins in Group V. In Group VI, all ACTINs contained Motif 1 and Motif 2, except for ItfACTIN18.2 (which lacked Motif 1) (Figure 3a).

The ACTIN domain (PF00022) acts as a key structure for the formation of filaments. Almost all of the ACTIN domains in different IbACTINs contained Motifs 1–8, except for IbACTIN12.3 (which lacked Motif 1 and Motif 8), IbACTIN5.4 (which lacked Motif 1), and IbACTINs in Group VI, which lacked Motif 1 (Figure 3b). Moreover, all of the ACTINs contained the ACTIN domain and only ItfACTIN19.1, -19.2, ItbACTIN19.1, -19.2, -19.3, and IbACTIN19 contained an F-box domain, which can regulate a variety of life activities, such as delaying plant senescence, regulating plant flowering, and responding to biotic stress, drought, and salt stress [35] (Figure 3b).

To better understand the structural diversity among the *ACTINs*, the exon-intron structures were analyzed (Figure 3c). The number of exons in the *ACTINs* ranged from 1 to 22. In more detail, the *ACTINs* of Group I contained 19 to 22 exons; the *ACTINs* of Group II contained 6 or 7 exons; the *ACTINs* of Group III and Group IV contained 1 to 6 exons, the *ACTINs* of Group V contained 6 to 16 exons, and the *ACTINs* of Group VI contained 14 to 19 exons (Figure 3c). The exon-intron structures of some homologous *ACTINs* were different in *I. batatas* compared with those in *I. trifida* and *I. triloba*, such as *IbACTIN17.3* (containing 6 exons), *ItfACTIN17.3* (containing 4 exons), and *ItbACTIN17.3* (containing 4 exons) in Group III; *IbACTIN5.6* (containing 4 exons), *ItfACTIN5.6* (containing 4 exons), and *ItbACTIN5.6* (containing 2 exons) in Group IV; and *ItfACTIN12.1* (containing 15 exons) and *IbACTIN12.1* (containing 16 exons) in Group V (Figure 3c). These results indicated that the *ACTIN* family may have undergone a lineage-specific differentiation event in the sweet potato genome.

### 2.4. Analysis of cis-Elements in the Promoter of IbACTINs in the Sweet Potato

**Figure 4:**
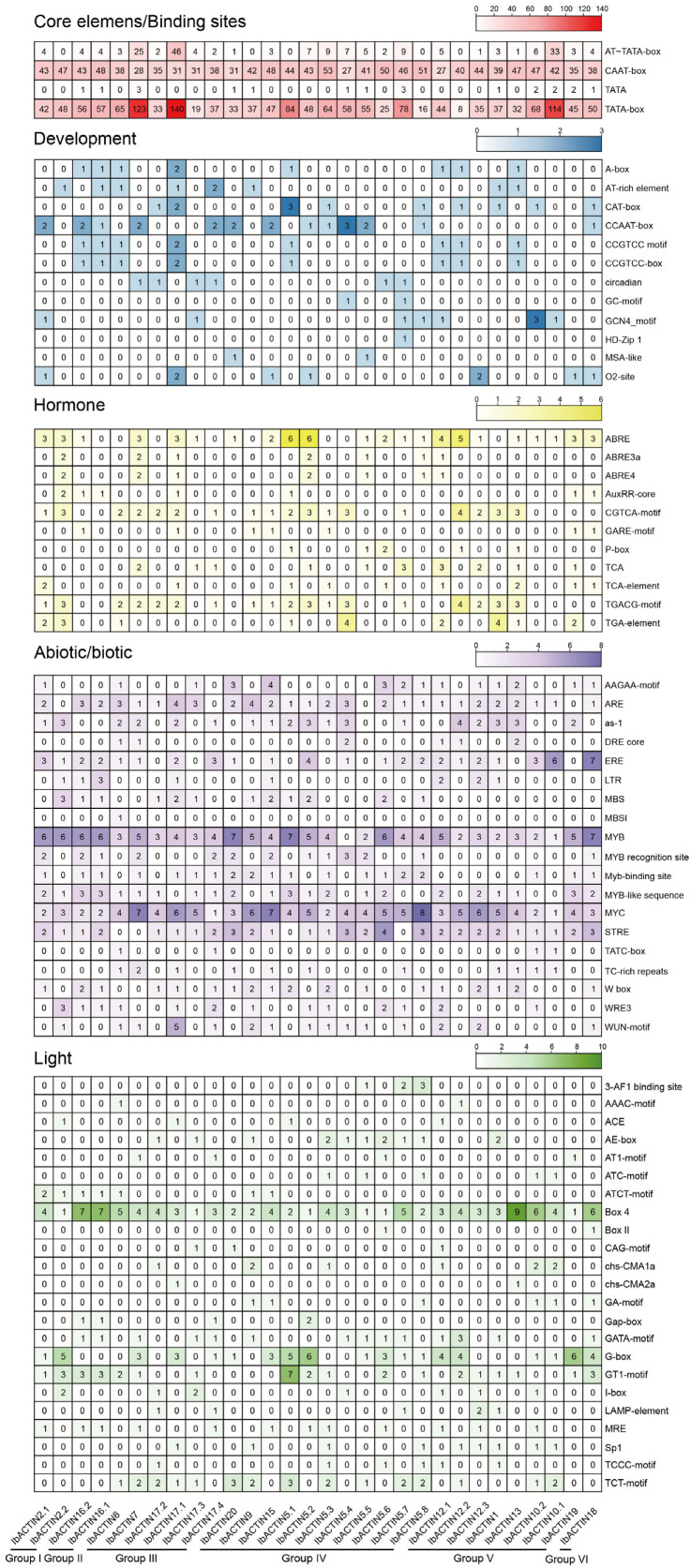
*cis*-element analysis of *IbACTINs* in *I. batatas*. The *cis*-elements were divided into five categories. The intensity of the different colors represents the number of *cis*-elements in the *IbACTIN* promoters.

Promoter *cis*-elements in plants initiate the gene functions related to plant development, hormone regulation, and stress responses. Therefore, we performed an analysis of the *cis*-elements using the 2000 bp promoter region of *IbACTINs*. According to the predicted functions, we divided the elements into five categories: core elements and binding sites, elements of developmental regulation, hormone-responsive elements, abiotic/biotic stress-responsive elements, and light-responsive elements (Figure 4). A large number of core elements were identified in 30 *IbACTINs* (CAAT-box and TATA-box) (Figure 4). Most of the *IbACTINs* contained several development elements, such as the O2 site, which is a zein metabolism regulatory element (found in *IbACTIN2.1*, *-17.1*, *-15*, *-5.2*, *-12.3*, *-19*, and *-18*); the CAT-box, which is associated with meristem formation (found in *IbACTIN17.1*, *-17.2*, *-5.1*, *-5.3*, *-5.8*, *-12.2*, *-1*, *-10.2*, and *-18*); and the GCN4 motif, which is involved in controlling seed-specific expression (found in *IbACTIN2.1*, *-17.3*, *-5.7*, *-5.8*, *-12.1*, *-10.2*, and *-10.1*) (Figure 4). Moreover, light-responsive elements such as the G-box, BOX4, and AE-box were abundant in the promoters of *IbACTINs* (Figure 4).

Additionally, some abiotic elements, such as the drought-responsive elements DRE-core, MYB, and MYC; the salt-responsive elements LTR, MBS, and W-box; the light-responsive elements ERE and LTR; and biotic elements such as WRE3, W-box, and the WUN motif, were identified in most *IbACTINs* (Figure 4). All the *IbACTINs* possessed several hormone elements, including ABRE for the ABA-responsive elements, TGA-element for the IAA-responsive elements, TATC-box for the GA-responsive elements, the CGTCA and TGACG motifs for the MeJA-responsive elements, and the TCA motif for the SA-responsive elements (Figure 4). These results suggest that *IbACTINs* are involved in the regulation of plant growth and development, hormone crosstalk, and abiotic stress adaptations in the sweet potato.

### 2.5. Protein Interaction Network of ACTINs in the Sweet Potato

**Figure 5:**
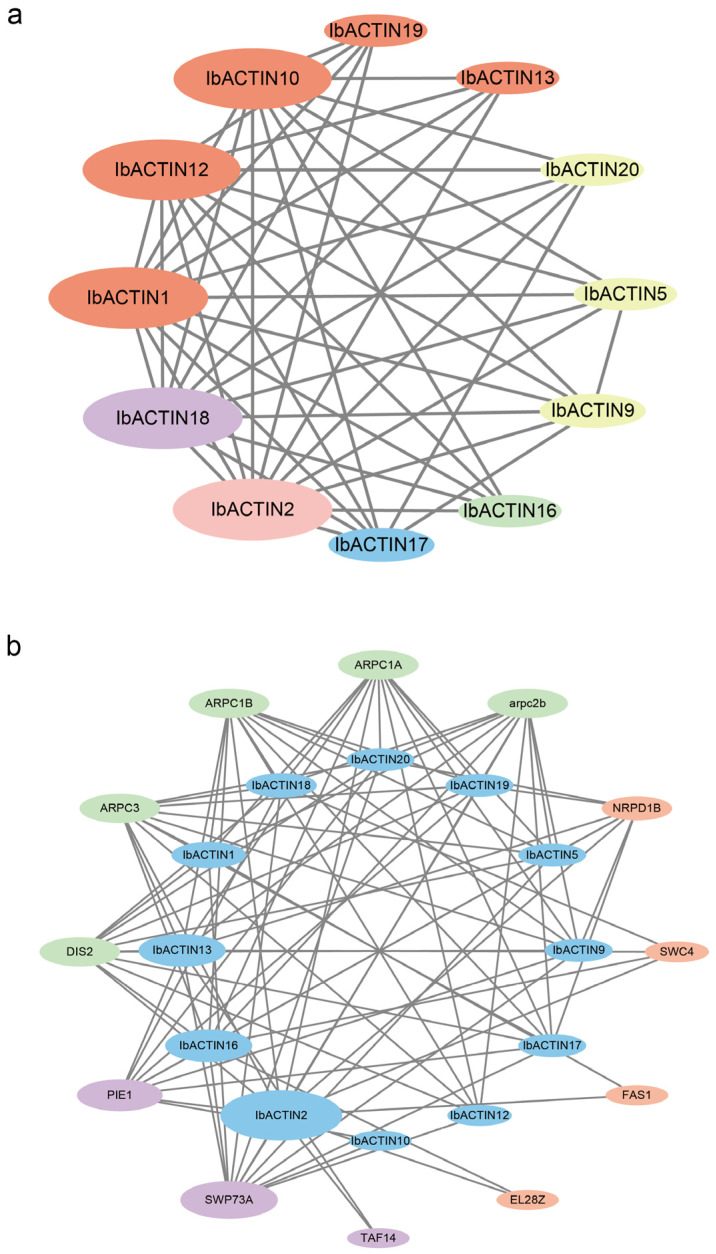
Functional interaction networks of IbACTINs in *I. batatas* according to their orthologs in *Arabidopsis thaliana*. The network nodes represent proteins, and the lines represent protein-protein associations. (**a**) The pink node, green node, blue node, yellow node, orange node, and purple node represent the IbACTINs in Group I, Group II, Group III, Group IV, Group V, and Group VI, respectively. The size of each node represents the number of proteins that interact with each other. The lines represent the interaction among ACTIN proteins. (**b**) The green node, orange node, and purple node represent the cell polarity development proteins, DNA transcription and translation proteins, and regulation of flower development proteins, respectively. The lines represent the interactions of the ACTINs and other proteins.

To explore the potential regulatory network of IbACTINs, we constructed an interaction network of IbACTINs based on the orthologous proteins from *Arabidopsis thaliana* (Figure 5). The predicted protein interactions indicated that IbACTINs could interact with each other to form heterodimers (Figure 5a). In addition, ACTINs can interact with the proteins of cell polarity development (i.e., Arpc2b [36], D1s2 [37], Arpc1b, Arpc1a, and Arpc3 [38]), and the regulation of flower development (i.e., PIE1 [39], Taf14 [40], and SWP73A [41]), DNA-directed RNA polymerase V subunit 1 Nrpd1b [42], the chromatin assembly factor Fas1 [43], DNA methyltransferase 1-associated protein SWC4 [44], and the phosphatidylinositol kinase family protein EL28Z [45] (Figure 5b). These results indicated that IbACTINs might participate in plant growth through interacting with related transcription factors and functional proteins.

### 2.6. Expression Analysis of ACTINs in the Sweet Potato and Its Two Diploid Relatives

#### 2.6.1. Analysis of Expression in Various Tissues

**Figure 6:**
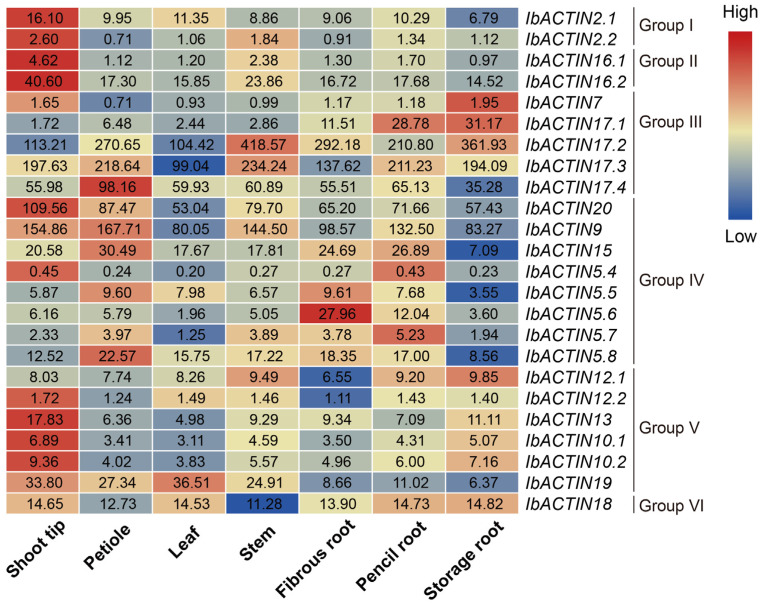
Gene expression patterns of the *IbACTINs* of Xushu18 in different tissues of *I. batatas* (shoot tip, petiole, leaf, stem, fibrous root, pencil root, and storage root).

To investigate the potential biological function of *IbACTINs* in plant growth and development, the expression levels in seven representative tissues (i.e., shoot tip, petiole, leaf, stem, fibrous root, pencil root, and storage root) of *I. batatas* were analyzed using the data obtained in the laboratory (Figure 6). In general, the expression of some *IbACTINs* in various tissues of Xushu18 was relatively stable, especially *IbACTIN12.1* (varying from 6.55 to 9.85), *-12.2* (varying from 1.11 to 1.72), and *-18* (varying from 11.28 to 14.82).

The different subgroups exhibited diverse expression patterns in the seven tissues, and different *IbACTINs* in the same subgroup exhibited regular expression patterns. Among all the *IbACTINs*, four *IbACTINs* (i.e., *IbACTIN17.2*, *-17.3*, *-20*, and *-9*) were highly expressed in all tissues. Interestingly, most *IbACTINs* showed high expression levels in the shoot tip. Moreover, some *IbACTINs* showed tissue-specific expression patterns. For example, *IbACTIN17.4*, *-9*, *-15*, *-5.5*, and *-5.8* were highly expressed in the petiole; *IbACTIN2.1* and *-19* were highly expressed in the leaves; *IbACTIN17.2* and *-17.3* were highly expressed in the stem; *IbACTIN5.5* and *-5.6* were highly expressed in fibrous roots; *IbACTIN17.1*, *-5.4*, and *-5.7* were highly expressed in pencil roots; and *IbACTIN7*, *-17.1*, *-17.2*, and *-12.1* were highly expressed in storage roots (Figure 6). These results indicated that *IbACTINs* may have different functions in different tissues of sweet potato and exhibit relatively stable expression patterns.

**Figure 7:**
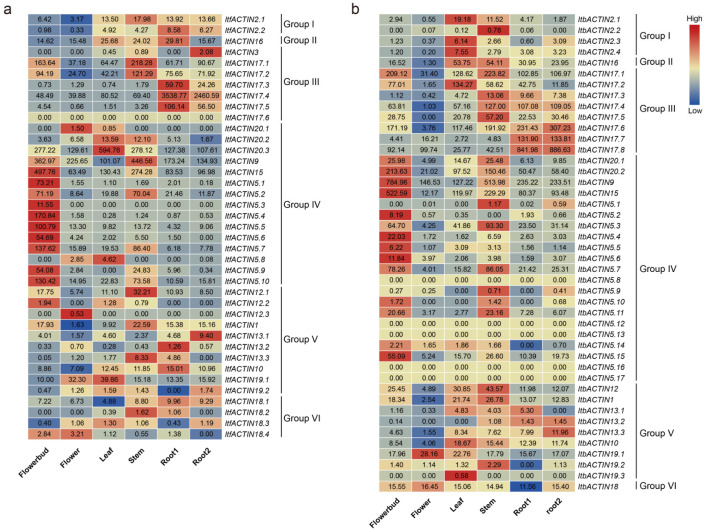
Gene expression patterns of *ItfACTINs* (**a**) and *ItbACTINs* (**b**) in the flower bud, flower, leaf, stem, root1, and root2 of *I. trifida* and *I. triloba*, as determined by RNA-seq. The log_2_ (FPKM+1) values are shown in the boxes.

In addition, we used the RNA-seq data from six tissues (i.e., flower bud, flower, leaf, stem, root1, and root2) to study the expression patterns of *ACTINs* in *I. trifida* and *I. triloba* [46] (Figure 7a,b). In *I. trifida, ItfACTIN20.3*, *-9*, and *-15* were highly expressed in all tissues. The *ItfACTINs* in Group IV were highly expressed in the flower buds, except for *ItfACTIN20.1*, *-20.2*, *-20.3*, and *-5.8*; *ItfACTIN20.1*, *-12.3*, *-19.1*, and *-18.4* were highly expressed in the flowers; *ItfACTIN20.2*, *-20.3*, *-5.8*, and *-19.1* were highly expressed in the leaves; *ItfACTIN17.1*, *-17.2*, *-9*, *-12.1*, *-1*, *-13.3*, and *-18.2* were highly expressed in the stems; *ItfACTIN2.2*, *-17.3*, *-17.4*, and *-17.5* were highly expressed in the roots (Figure 7a). In *I. triloba*, the expression levels of *ItbACTIN18* (varying from 11.56 to 16.45) were relatively stable and *ItbACTIN9* was highly expressed in all tissues. Over half *ItbACTINs* in Group IV were highly expressed in the flower buds; *ItbACTIN19.1* was highly expressed in the flowers; *ItbACTIN2.1*, *-2.3*, *-2.4*, *-17.2*, and *-19.3* were highly expressed in the leaves; most *ItbACTINs* were highly expressed in the stems; and *ItbACTIN17.6*, *-17.7*, *-17.8*, *-13.2*, and *-13.3* were highly expressed in the roots (Figure 7b). These results showed that *ACTINs* exhibit different expression patterns and play important roles in the growth and development of the sweet potato and the two diploids.

#### 2.6.2. Analysis of Expression in Different Developmental Stages

**Figure 8:**
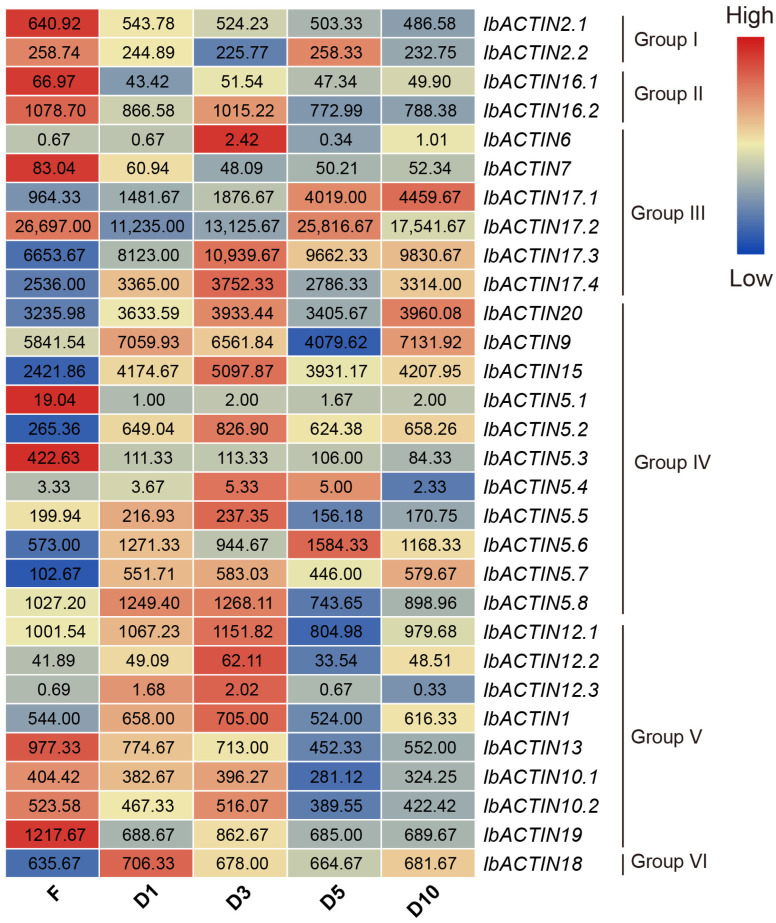
Gene expression patterns of *IbACTINs* in different developmental stages of the root as determined by RNA-seq. F, fibrous root (diameter of approximately 1 mm); D1, initial storage root (diameter of approximately 1 cm); D3, storage root (diameter of approximately 3 cm); D5, storage root (diameter of approximately 5 cm); D10, storage root (diameter of approximately 10 cm).

The plant morphology of the cultivated hexaploid sweet potato is different from that of its diploid relatives, especially since the diploid relatives cannot form tuberous roots. To comprehensively identify all *IbACTINs* in different developmental tuberous, we also used the RNA-seq data to evaluate the expression levels of *IbACTINs* in different developmental stages of sweet potato roots (i.e., at F, D1, D3, D5, and D10) (Figure 8) [47].

Notably, the expression levels of *IbACTINs* were relatively stable in the five stages of root development, especially *IbACTIN2.1* (varying from 486.58 to 640.92), *-2.2* (varying from 225.77 to 258.33), *-16.1* (varying from 43.42 to 66.97), *-16.2* (varying from 772.99 to 1078.70), *-20* (varying from 3235.98 to 3960.08), *-5.8* (varying from 743.65 to 1268.11), *-12.1* (varying from 804.98 to 1151.82), *-10.2* (varying from 389.55 to 523.58), and *-18* (varying from 635.67 to 706.33). It is worth noting that the expression levels of 21 *IbACTINs* were significantly higher than those of the other *IbACTINs* at all stages, especially *IbACTIN17.1*, *-17.2*, *-17.3*, *-17.4*, *-20*, *-9*, and *-15.* However, some *IbACTINs* expressions had a certain specificity, e.g., *IbACTIN7*, *-5.1*, *-5.3*, *-13*, *-10.1*, and *-19* showed significantly higher expression levels in the fibrous roots (with a diameter of approximately 1 mm) than in other developmental stages. Moreover, *IbACTIN6*, *15*, *-5.2*, *-5.5*, *-12.2*, *-12.3*, and *-1* showed higher expression levels in the D3 stage; *IbACTIN17.1*, *-17.2*, and *-5.6* showed higher expression levels in the D5 stage; and *IbACTIN17.1*, *-17.3*, *-17.4*, and *-9* were highly expressed in the D10 stage. These results indicated that *IbACTINs* were of vital importance to the growth and development of tuberous roots in the sweet potato.

#### 2.6.3. Analysis of the Expression of Hormone Response

To investigate the potential biological functions of *ItfACTINs* and *ItbACTINs* in the hormone signal transduction and crosstalk of plants, we investigated the expression patterns of *ACTINs* under various hormonal treatments in order to explore the relationships between *ACTINs* and hormones. We analyzed the expression patterns of *ItfACTINs* and *ItbACTINs* using the RNA-seq data of *I. trifida* and *I. triloba* under treatments with ABA, IAA, GA, and BAP [33].

Most *ACTINs* of *I. trifida* and *I. triloba* were relatively stable under the treatment of various hormones, especially *ItfACTIN2.2* (varying from 5.94 to 7.38), *-16* (varying from 32.72 to 44.86), *-5.5* (varying from 6.17 to 8.36), *-13.3* (varying from 6.95 to 8.89), *-10* (varying from 12.57 to 16.45); *ItbACTIN2.1* (varying from 10.27 to 13.94), *-2.4* (varying from 3.12 to 3.90), *-17.5* (varying from 11.17 to 14.71), *-15* (varying from 77.33 to 109.43), *-12* (varying from 12.82 to 17.93), *-1* (varying from 11.15 to 15.93), *-19.1* (varying from 31.11 to 36.13), and *-18* (varying from 11.73 to 19.07). At the same time, the gene expression levels of some *ACTINs* under different hormone treatments were different, especially *ItfACTIN5.7* (varying from 6.16 to 28.68), *ItfACTIN5.9* (varying from 1.01 to 11.48), *ItbACTIN17.7* (varying from 3.87 to 39.32), and *ItbACTIN17.8* (varying from 54.83 to 122.65).

**Figure 9:**
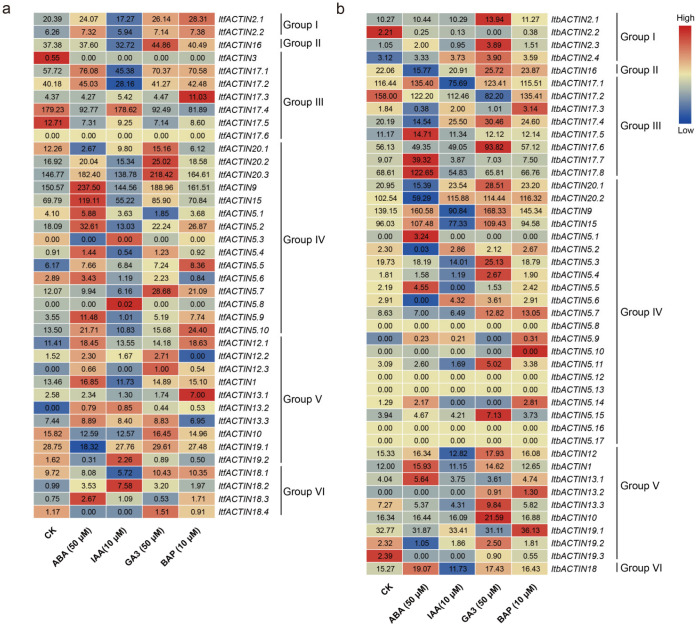
Gene expression patterns of *ACTINs* in response to different phytohormones (ABA, GA, IAA, and BAP) in *I. trifida* (**a**) and *I. triloba* (**b**) as determined by RNA-seq. The log2 (FPKM+1) values are shown in the boxes.

In *I. trifida*, *ItfACTIN9*, *-15*, *-5.2*, *-5.9*, and *-18.3* were highly induced by ABA, and *ItfACTIN19.2* and *-18.2* were induced by IAA. *ItfACTIN16*, *-20.2*, *-20.3*, *-5.7*, *-12.2*, and *-12.3* were highly induced by GA3. *ItfACTIN17.3*, *-5.10*, *-12.1*, and *-13.1* were highly induced by BAP. *ItfACTIN5.5*, *-12.1*, *-13.2*, and *-18.2* were induced by all the hormones, but *ItfACTIN3*, *-17.4*, and *-17.5* were repressed by all the hormones (Figure 9a).

In *I. triloba*, *ItbACTIN17.7*, *-17.8*, *-5.1*, and *-5.5* were highly induced by ABA. *ItbACTIN5.6* was induced by IAA. *ItbACTIN2.3*, *-17.4*, *-17.6*, *-5.3*, *-5.4*, *-5.11*, *-5.15*, and *-10* were highly induced by GA3. *ItbACTIN17.3*, *-5.7*, *-5.9*, *-5.14*, and *-13.2* were induced by BAP (Figure 9b). *ItbACTIN2.1* and *ItbACTIN2.4* were induced by all the treatments, but *ItbACTIN2.2*, *-17.2*, and *-19.3* were repressed under all the hormone treatments. These results indicated that *ACTINs* are involved in different hormonal pathways in the sweet potato and its two diploid relatives (Figure 9a,b).

#### 2.6.4. Analysis of Expression under Abiotic Stresses

**Figure 10:**
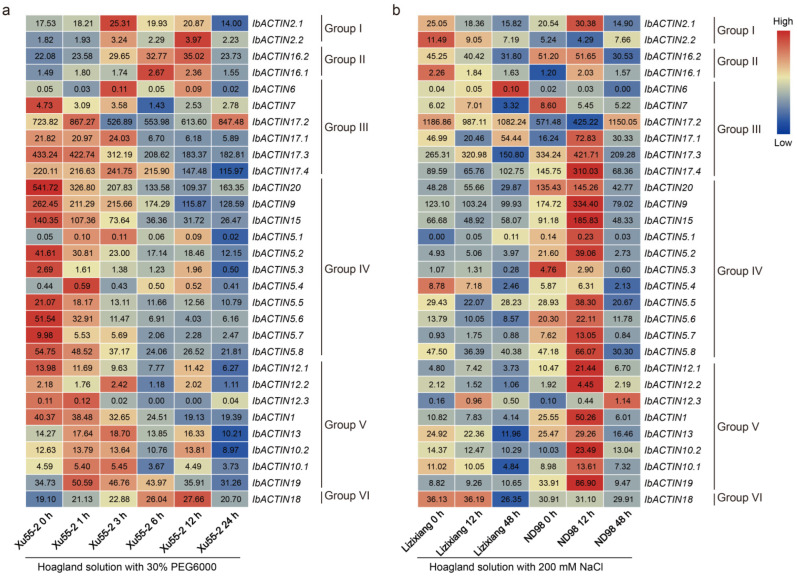
Gene expression patterns of *IbACTINs* under drought and salt stresses as determined by RNA-seq. (**a**) Expression of *IbACTINs* under PEG treatment in a drought-tolerant variety, i.e., Xu55-2. (**b**) Expression of *IbACTINs* under NaCl treatment in a salt-sensitive variety, i.e., Lizixiang, and a salt-tolerant line, i.e., ND98. The log_2_ (FPKM) values are shown in the boxes.

To explore the possible roles of *IbACTINs* in response to an abiotic stress, we analyzed the expression patterns of *IbACTINs* using the RNA-seq data of a drought-tolerant variety (Xu55-2) under drought stress, and the RNA-seq data of a salt-sensitive variety (Lizixiang) and a salt-tolerant line (ND98) under salt stress [48,49]. Most *ACTINs* were inhibited by PEG and NaCl treatments in Xu55-2 and Lizixiang, while most *ACTINs* were induced by NaCl treatments in ND98. It is noteworthy that some *ACTINs* were still relatively stable under PEG and NaCI treatment, e.g., *IbACTIN16.2* (varying from 22.08 to 35.02 under PEG; varying from 30.53 to 51.20 under NaCI) and *IbACTIN18* (varying from 19.10 to 27.66 under PEG; varying from 26.35 to 36.19 under NaCI). Moreover, *IbACTIN5.4* (varying from 0.43 to 0.59), *-10.2* (varying from 8.97 to 13.81), and *-10.1* (varying from 3.67 to 5.45) were stably expressed under PEG. *IbACTIN16.2* (varying from 31.80 to 45.25), *-17.2* (varying from 987.11 to 1186.86), *-9* (varying from 99.93 to 123.10), *-15* (varying from 48.92 to 66.88), *-5.2* (varying from 3.97 to 5.06), *-5.6* (varying from 8.57 to 13.79), *-10.2* (varying from 10.29 to 14.37), and *-19* (varying from 8.82 to 10.65) in Lizixiang, and *IbACTIN7* (varying from 5.22 to 8.60) in ND98 were stably expressed under NaCl treatment (Figure 10).

Among all the *ACTINs*, *IbACTIN2.1*, *-2.2*, *-16.1*, *-16.2*, *-6*, *-5.1*, and *-18* were induced by the PEG treatment in Xu55-2, and *IbACTIN6*, *-17.3*, *-20*, *-5.1*, *-5.7*, *-12.1*, and *-12.3* were induced by NaCl treatment in Lizixiang, whereas most *IbACTINs* from the salt-tolerant variety ND98 were induced under salt treatment, except for *IbACTIN2.2*, *-7*, *-17.2*, and *-5.3.* Meanwhile, the gene expression levels of some *ACTINs* under different abiotic stresses were different, especially *IbACTIN20* (varying from 109.37 to 541.72 under PEG), *IbACTIN5.6* (varying from 4.03 to 51.54 under PEG), *IbACTIN5.2* (varying from 2.73 to 39.06 under NaCI), and *IbACTIN19* (varying from 8.82 to 86.90 under NaCI).

**Figure 11:**
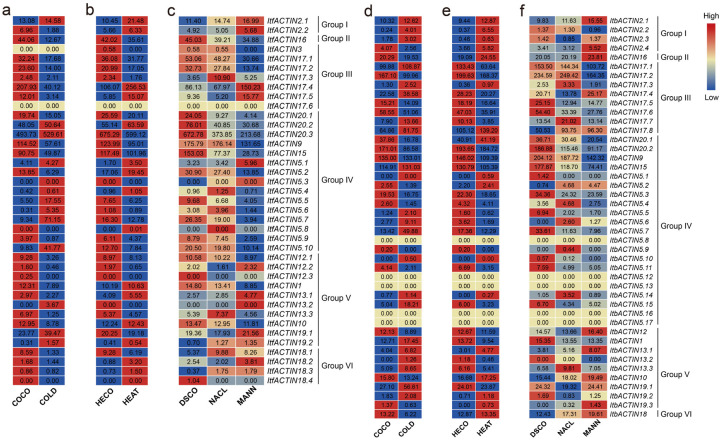
(**a**–**c**) Gene expression patterns of *ACTINs* under abiotic stresses (cold, heat, salt, and drought) in *I. trifida*, as determined by RNA-seq. (**d**–**f**) Gene expression patterns of *ACTINs* under abiotic stresses (cold, heat, salt, and drought) in *I. triloba*, as determined by RNA-seq. COCO, COLD, HECO, HEAT, DSCO, NACL, and MANN represent the cold control at 28/22-deg C day/night experiment, cold stress at 10/4-deg C day/night experiment, heat control at 28/22-deg C day/night experiment, heat stress at 35/35-deg C day/night experiment, drought and salt control, NaCl salt stress experiment, and mannitol drought stress experiment, respectively. The log_2_ (FPKM+1) values are shown in the boxes.

In addition, we also analyzed the expression patterns of *ACTINs* using the RNA-seq data of *I. trifida* and *I. triloba* under cold, heat, drought, and salt stress treatments [33]. Some *ACTINs* of *I. trifida* and *I. triloba* were relatively stable under various abiotic stresses. In *I. trifida*, under cold and heat treatment, compared with the control, *ItfACTIN20.2* (varying from 48.05 to 63.59) and *-20.3* (varying from 493.73 to 675.29) were stable (Figure 11a,b). Under the salt and drought stress treatments, *ItfACTIN2.1* (varying from 11.40 to 16.99), *-2.2* (varying from 4.92 to 5.68), *-16* (varying from 34.68 to 45.03), *-9* (varying from 131.65 to 176.14), *-12.1* (varying from 8.97 to 10.58), *-12.2* (varying from 1.61 to 2.32), *-10* (varying from 11.81 to 13.47), and *-19.1* (varying from 17.93 to 21.56) were stable (Figure 11c).

In *I. triloba*, *ItbACTIN2.1* (varying from 9.44 to 12.87), *-16* (varying from 19.09 to 24.55), *-17.5* (varying from 14.09 to 18.19), *-9* (varying from 109.39 to 146.02), *-15* (varying from 105.39 to 131.03), *-5.3* (varying from 16.75 to 22.30), *-12* (varying from 8.89 to 12.67), and *-10* (varying from 13.24 to 17.25) showed stable expression patterns under cold and heat stresses compared with the control (Figure 11d,e). *ItbACTIN2.2* (varying from 0.96 to 1.30), *-16* (varying from 20.05 to 23.81), *-9* (varying from 142.32 to 204.12), *-5.15* (varying from 4.34 to 6.70), *-12* (varying from 13.66 to 16.40), *-1* (varying from 13.35 to 15.35), *-13.3* (varying from 6.58 to 9.81), *-10* (varying from 15.44 to 19.49), and *-19.1* (varying from 19.32 to 24.41) showed stable expression patterns under the salt and drought stress treatments (Figure 11f). These results indicated that *ACTINs* showed commonalities and differences in their responses to abiotic stresses in *I. trifida* and *I. triloba*.

## 3. Discussion

ACTINs are structural proteins widely distributed in plants. They are the main components of microfilaments and participate in many crucial physiological activities including the maintenance of cell shape and cytoplasmic streaming [1,2,3,4,5,6]. However, the functions and transcriptional regulatory mechanisms of ACTINs remain largely unknown in the sweet potato. As the genetic background of the cultivated sweet potato is complex, previous studies on the gene families of the sweet potato have mainly focused on its most probable progenitor diploids [50,51,52,53]. In fact, the plant morphology of the cultivated hexaploid sweet potato differs greatly from that of its diploid relatives, especially since its diploid relatives cannot form tuberous roots [33]. In this study, we systematically identified *ACTIN* family genes, and analyzed and compared their characteristics on the basis of the draft genome sequence of the cultivated hexaploid sweet potato and its two diploid relatives. This genome-wide study of *ACTINs* may play an important guiding role in the further study of their function and in the molecular breeding of the sweet potato.

### 3.1. Evolution of the ACTIN Gene Family in the Sweet Potato and Its Two Diploid Relatives

In this study, 113 ACTINs were identified in the sweet potato and its two diploid relatives. The number of ACTINs identified in *I. batatas* (30) was nine less than that in *I. trifida* (39), but 14 less than that in *I. triloba* (44) (Figure 1; Table S1). Genomic alignment revealed the differentiation and evolution of the chromosomes [54]. The chromosome localization and distribution of the *ACTINs* in each chromosome differed among *I. batatas*, *I. trifida*, and *I. triloba*; 12 chromosomes contained *ACTIN* genes in *I. trifida* and *I. triloba*, but 13 chromosomes contained *ACTIN* genes in *I. batatas* (Figure 1). On the basis of the phylogenetic relationships, the ACTINs were divided into six subgroups (Groups I to VI). Moreover, the number and type of ACTINs distributed in each subgroup of sweet potato and its two diploid relatives were different from those in *Arabidopsis thaliana* (Figure 2). These results revealed that the *ACTIN* gene family might have undergone a lineage-specific differentiation event in the terrestrial plant genome.

Eight conserved motifs were identified in most ACTINs, and all the ACTINs contained an ACTIN domain, indicating that these motifs were relatively conserved in the evolution of the sweet potato and its two diploid relatives (Figure 3b). Introns usually act as buffer zones or mutation-resistant fragments that reduce adverse mutations and insertions. Moreover, introns also play essential roles in the export of mRNA, transcriptional coupling, alternative splicing, the regulation of gene expression, and other biological processes [54,55]. Here, the exon-intron distributions of some homologous *ACTINs* were different in *I. batatas* compared with those in *I. trifida* and *I. triloba* (Figure 3c). For example, in Group III, *IbACTIN17.3* contained six introns, but its homologous genes, *ItfACTIN17.3* and *ItbACTIN17.3* contained four introns. Their expression levels in various tissues showed differences. For example, *IbACTIN17.3* was highly expressed in various tissues while *ItfACTIN17.3* and *ItbACTIN17.3* showed lower expression levels. In the sweet potato and the two diploids, these differences in the exon-intron structure may result in the different functions carried out by the *ACTINs* during growth and development.

### 3.2. Stable Expression of ACTIN in the Sweet Potato and Its Two Diploid Relatives

The ideal internal reference gene should stably be expressed in different development stages and different tissues and organs, not be subject to cell cycle regulation, and not be affected by endogenous and exogenous signals such as temperature, light, biotic or abiotic stress [56,57]. *ACTINs*, as housekeeping genes, are widely used as internal reference genes [25,26,27,28]. Since several *IbACTINs* were expressed stably under various treatments, we selected *IbACTINs* as internal reference genes. Collectively, *IbACTIN18* is stably expressed in different tissues and treatment, indicating that it could be widely used as the internal reference gene in sweet potatoes. During root developmental stages, *IbACTIN20* could be used as the internal reference gene. In tissue-specific expression experiments, *IbACTIN17.2*, *-17.3*, *-20* and *-9* could be used as internal reference genes. In salt-drought stress experiments, *IbACTIN16.2* and *-18* could be used as internal reference genes. (Figure 10). In *I. trifida* and *I. triloba*, *ItfACTIN18.3* and *ItbACTIN18* could be used as internal reference genes in tissue-specific expression experiments. Some *ACTINs* (*ItfACTIN2.2*, *-16*, *-5.5*, *-13.3*, and *-10*; and *ItbACTIN2.1*, *-2.4*, *-17.5*, *-15*, *-12*, *-1*, *-19.1*, and *-18)* could be used as internal reference genes in hormone treatment experiments and *Itf/ItbACTIN2.2*, *-16*, *-9*, *-10*, and *-19.1* could be used as internal reference genes in salt and drought treatment experiments. These results indicated that these *ACTIN* substances may serve as internal reference genes in different experiments.

### 3.3. Differences in the Functions of ACTINs in Growth and Development between the Sweet Potato and Its Two Diploid Relatives

ACTINs have been reported to perform relatively basic functions in plants, and different genes in the family have specific functions. In *Arabidopsis thaliana*, ACT7 not only plays an important role in callus formation, but also responds strongly to external stimuli [58]. Moreover, the genes *ACT12* and *ACT4* are mainly expressed in the process of pollen tube elongation and may play an important role in this process [59]. The genes *PEAcI* and *PEAcII* in the pea contain regulatory sequences which can adapt to the needs of plant growth at different stages and fine-tune their expression [60]. For sweet potatoes, it is crucial to identify the most suitable reference gene for gene studies. ACTIN, as the basic component of the organelle skeleton necessary for the life-sustaining activities of organisms, is not only stably expressed in various cells and physiological states, but is also the basis for normal cell growth, and it may play an important role in the growth and development of the sweet potato [3].

To further explore the functions of *ACTINs* in growth and development, we analyzed the predicted *cis*-elements. *IbACTIN17.1* and *-17.2*, which contained the meristem formation and cell division-related element CAT-box, were highly expressed in all the tissues, indicating that *IbACTIN17.1* and *-17.2* may play regulatory roles in the development of sweet potatoes. The predicted protein interactions showed that IbACTINs interacts with the transcriptional relative protein (1Nrpd1b, Fas1, and SWC4) [42,43,44], indicating that IbACTINs may play crucial roles in the transcriptional process. These results suggested that IbACTINs may participate in plant growth by interacting with transcription factors and functional proteins related to cell division.

### 3.4. Different Functions of ACTINs in Hormone Crosstalk in the Sweet Potato and Its Two Diploid Relatives

ACTINs have been reported to participate in the regulation of multiple hormones. ACT7 plays a central role in maintaining optimal root elongation through regulating ACTIN’s dynamicity and the abundance of PIN1 and PIN2, which are linked to the intracellular auxin homeostasis regulated by the ACT7 of the vegetative class [61]. The ACTIN isovariant ACT7 mediates the redistribution of auxin in root tips by regulating the auxin-ethylene reaction, thus controlling the development of the main root’s meristem [62].

In this study, most *IbACTIN* genes were induced by at least one hormone, except for *IbACTIN16.1*, whose promoter only contained an IAA-responsive element (AuxRR-core). Due to the differences in gene structures and motifs, the *ACTINs* in the two diploids exhibited different expression patterns; for example, *ItbACTIN2.2* was repressed by ABA, IAA, GA3, and BAP treatments, but *ItfACTIN2.2* was induced by ABA, GA3, and BAP treatments. These results indicated that *ACTINs* are involved in the crosstalk of multiple hormones, and those homologous *ACTIN* genes participate in different hormone pathways in the sweet potato and its two diploid relatives (Table S2). However, the roles of *ACTINs* in the regulation of hormone crosstalk need further investigation.

### 3.5. Different Functions of ACTINs in Abiotic Stress Responses in the Sweet Potato and Its Two Diploid Relatives

ACTINs have been reported to participate in the abiotic stress response in plants. ACTIN filaments take center stage in stress-induced signaling pathways, either as a direct target or as a signal transducer [63]. The analysis of the predicted *cis*-elements showed that some abiotic elements, such as the drought-responsive elements MYB and MYC, and salt stress-responsive elements ABRE and W-box, were identified in all *IbACTINs* (Figure 4). Most *ACTINs* were inhibited by the PEG treatments in Xu55-2, while most *ACTINs* were induced by the NaCl treatments in ND98. In the two diploid relatives, some *ACTINs* (*ItfACTIN2.1*, *-17.3*, *-19.2*, and *-18.3* and *ItbACTIN2.1*, *-5.2*, *-13.1*, *-13.3*, *-10*, *-19.3*, and *-18*) were induced by both the drought and salt treatments (Figure 11). These ACTINs may serve as candidate genes for use in improving abiotic stress tolerance in the sweet potato.

## 4. Materials and Methods

### 4.1. Identification of ACTINs

The whole genome sequence of *I. batatas*, *I. trifida* and *I. triloba* were downloaded from *Ipomoea* Genome Hub (https://ipomoea-genome.org/, accessed on 8 January 2023) and the Sweetpotato Genomics Resource (http://sweetpotato.plantbiology.msu.edu/, accessed on 8 January 2023). To accurately identify all ACTIN family members, three different screening methods were combined. Firstly, the BLAST algorithm was used to identify predicted ACTINs using all AtACTINs from the *Arabidopsis thaliana* genome database (https://www.arabidopsis.org/, accessed on 8 January 2023) as queries (BLASTP, E value ≤ 1 × 10^−5^). Next, the HMMER 3.0 software (Harvard University, Cambridge, MA, USA) was used to identify potential ACTINs through the Hidden Markov Model profiles (hmmsearch, E value ≤ 1 × 10^−5^) of the ACTIN domain (PF00022), which were extracted from the Pfam databases (http://pfam.xfam.org/, accessed on 8 January 2023). Finally, all putative ACTINs were ensured using SMART (http://smart.embl-heidelberg.de/, accessed on 8 January 2023) and CD-search (https://www.ncbi.nlm.nih.gov/Structure/cdd/wrpsb.cgi, accessed on 8 January 2023).

### 4.2. Chromosomal Distribution of ACTINs

The *IbACTINs*, *ItfACTINs*, and *ItbACTINs* were separately mapped to the *I. batatas, I. trifida*, and *I. triloba* chromosomes, respectively, based on the chromosomal locations provided in the *Ipomoea* Genome Hub (https://ipomoea-genome.org/, accessed on 17 January 2023) and Sweetpotato Genomics Resource (http://sweetpotato.plantbiology.msu.edu/, accessed on 17 January 2023). The visualization was generated using the TBtools software (v1.098696) (South China Agricultural University, Guangzhou, China) [64].

### 4.3. Protein Properties Prediction of ACTINs

The MW, theoretical pI, unstable index, and hydrophilic of the ACTINs were calculated using ExPASy (https://www.expasy.org/, accessed on 20 January 2023). The phosphorylation sites of the ACTINs were predicted using GPS 5.0 [65]. The subcellular localization of the ACTINs was predicted using WoLF PSORT (https://wolfpsort.hgc.jp/, accessed on 20 January 2023).

### 4.4. Phylogenetic Analysis of ACTINs

Multiple sequence alignment of the deduced amino acid sequences of the ACTINs from *I. batatas*, *I. trifida*, *I. triloba*, and *Arabidopsis thaliana* were aligned with Clustal X, and the alignment was imported into MEGA11 to create a phylogenetic tree using the maximum likelihood method with 1000 bootstrap replicates (www.megasoftware.net, accessed on 30 January 2023) [66]. Then, the phylogenetic tree was constructed using iTOL (http://itol.embl.de/, accessed on 30 January 2023).

### 4.5. Domain Identification and Conserved Motifs Analysis of ACTINs

The conserved motifs of the ACTINs were analyzed using MEME software (v5.5.3) (https://meme-suite.org/meme/, accessed on 2 February 2023). The MEME parameters were set to search for a maximum of 8 motifs [67].

### 4.6. Exon-Intron Structures and Promoter Analysis of ACTINs

The exon-intron structures of the *ACTINs* were obtained from GSDS 2.0 (http://gsds.gao-lab.org/, accessed on 2 February 2023) and were visualized using the TBtools software (v1.098696). The *cis*-elements in the approximately 2000 bp promoter region of the *ACTINs* were predicted using PlantCARE (http://bioinformatics.psb.ugent.be/webtools/plantcare/html/, accessed on 2 February 2023) [68].

### 4.7. Protein Interaction Network of ACTINs

The protein interaction networks of the ACTINs were predicted using STRING (https://cn.string-db.org/, accessed on 5 February 2023) based on *Arabidopsis thaliana* homologous proteins. The network map was built using Cytoscape software (v3.9.1) [69,70,71].

### 4.8. Transcriptome Analysis

The RNA-seq data of *ItfACTINs* and *ItbACTINs* in *I. trifida* and *I. triloba* were downloaded from the Sweetpotato Genomics Resource (http://sweetpotato.plantbiology.msu.edu/, accessed on 7 February 2023). The RNA-seq data of *IbACTINs* in *I. batatas* were obtained from the NCBI SRA repository under the accession number SRP092215 [48,49]. The expression levels of the *ACTINs* were calculated as fragments per kilobase of exon per million fragments mapped (FPKM). The heat maps were constructed using the Tbtools software (v1.098696) [64].

## 5. Conclusions

In this study, we identified and characterized 30, 39, and 44 *ACTINs* in cultivated hexaploid sweet potato (*I. batatas*, 2n = 6x = 90) and its two diploid relatives, *I. trifida* (2n = 2x = 30) and *I. triloba* (2n = 2x = 30), respectively, on the basis of their genomic and transcriptomic data. The protein physicochemical properties, chromosome localization, phylogenetic relationships, gene structures, promoter *cis*-elements, and protein interaction networks of these 113 *ACTINs* were systematically investigated. Moreover, the tissue specificity and expression patterns of the *ACTINs* in the development of tuberous roots, hormone responses, and abiotic stress responses were analyzed using RNA-seq.

The results indicated that there was a difference in the functions of homologous *ACTINs* in the sweet potato and its two diploid relatives, and the expression patterns of some *ACTINs* were relatively constant under different treatments in different tissues. Each *ACTIN* gene played different vital roles in the plants’ growth and development, hormone crosstalk, and abiotic stress responses. Moreover, there were some suitable *ACTINs*, e.g., *IbACTIN18* (in various tissues and treatments); *IbACTIN20* (in various tissues); *IbACTIN16.2* (in roots, and under drought and salt treatments), *ItfACTIN2.2*, *-16*, and *-10* (under various treatments); *ItbACTIN18* (in various tissues and hormone treatments); and *ItbACTIN19.1* (under various treatments). These *ACTINs* can be picked as internal reference genes in different experiments. This study provides valuable insights into the structure and function of *ACTIN* genes in the sweet potato and its two diploid relatives.

## Figures and Tables

**Table 1:** Characterization of *IbACTINs* in the sweet potato.

Number	Gene ID	Gene Name	PI	Molecular Weight (kDa)	CDS Length (bp)	Phosphorylation Site			Protein Size (aa)	Aliphatic Index	Instability Index	GRAVY	Subcellular Locations	*Arabidopsis* Homologs
						Ser	Thr	Tyr						
1	g40378	*Ib* *ACTIN* *1*	5.17	36.83	1008	14	12	9	330	73.27	36.01	−0.268	Cytoplasm	*ACTIN* *1*
2	g5780	*IbACTIN2.1*	5.05	48.02	1329	24	12	7	435	84.97	39.93	−0.289	Nucleus	*ACTIN2*
3	g52926	*IbACTIN2.2*	6.98	58.59	1599	29	10	8	524	81.49	51.07	−0.355	Nucleus	*ACTIN2*
4	g4902	*IbACTIN5.1*	5.31	41.73	1152	17	6	8	377	84.08	35.25	−0.202	Cytoskeleton	*ACTIN5*
5	g40273	*IbACTIN5.2*	5.31	41.70	1152	16	7	8	377	84.62	34.16	−0.186	Cytoskeleton	*ACTIN5*
6	g9979	*IbACTIN5.3*	5.42	42.32	1167	17	10	7	382	84.27	33.16	−0.157	Cytoskeleton	*ACTIN5*
7	g18180	*IbACTIN5.4*	7.87	12.45	345	2	2	1	113	106.02	36.03	0.15	Cytoplasm	*ACTIN5*
8	g24983	*IbACTIN5.5*	5.31	41.73	1152	15	8	8	377	84.08	34.71	−0.206	Cytoskeleton	*ACTIN5*
9	g43021	*IbACTIN5.6*	5.47	40.56	1110	17	8	8	363	86.25	34.98	−0.211	Cytoplasm	*ACTIN5*
10	g3675	*IbACTIN5.7*	5.07	38.73	1065	16	5	7	349	82.12	34.98	−0.143	Cytoskeleton	*ACTIN5*
11	g24981	*IbACTIN5.8*	5.31	41.76	1152	17	8	8	377	83.55	35.22	−0.22	Cytoskeleton	*ACTIN5*
12	g52274	*IbACTIN6*	9.33	39.10	1053	26	8	6	345	79.88	55.76	−0.266	Chloroplast	*ACTIN6*
13	g51848	*IbACTIN7*	9.78	24.93	693	29	5	3	227	78.19	69.01	−0.406	Chloroplast	*ACTIN7*
14	g25621	*IbACTIN9*	5.38	41.68	1149	14	9	8	376	85.88	36.46	−0.178	Cytoskeleton	*ACTIN9*
15	g2351	*IbACTIN10.1*	5.68	73.78	1965	32	10	9	644	68.32	53.54	−0.738	Cytoplasm	*ACTIN10*
16	g2496	*IbACTIN10.2*	5.78	82.66	2196	35	11	9	720	68.83	54.01	−0.749	Cytoplasm	*ACTIN10*
17	g64096	*IbACTIN12.1*	6.11	57.53	1551	20	14	7	508	81.48	46.25	−0.305	Cytoplasm	*ACTIN12*
18	g48567	*IbACTIN12.2*	8.54	33.65	915	11	9	5	300	83.47	43.54	−0.202	Chloroplast	*ACTIN12*
19	g18621	*IbACTIN12.3*	9.05	28.13	771	18	10	4	252	80.91	53	−0.208	Nucleus	*ACTIN12*
20	g26691	*IbACTIN13*	6.43	57.52	1584	31	21	3	519	91.81	42.68	−0.125	Cytoplasm	*ACTIN13*
21	g58376	*IbACTIN15*	5.54	38.77	1065	16	8	7	349	86.1	36.11	−0.193	Cytoskeleton	*ACTIN15*
22	g42892	*IbACTIN16.1*	4.71	27.97	783	7	6	2	256	96.41	45.39	−0.026	Cytoplasm	*ACTIN16*
23	g63730	*IbACTIN16.2*	4.79	40.02	1116	11	10	5	365	91.37	45.53	−0.081	Cytoplasm	*ACTIN16*
24	g46627	*IbACTIN17.1*	5.1	41.61	1152	19	6	8	377	86.13	31.71	−0.154	Cytoskeleton	*ACTIN17*
25	g60046	*IbACTIN17.2*	5.31	41.74	1152	17	8	8	377	85.86	33.06	−0.162	Cytoskeleton	*ACTIN17*
26	g27691	*IbACTIN17.3*	5.42	36.18	996	15	4	5	326	89.45	42.1	−0.085	Cytoplasm	*ACTIN17*
27	g723	*IbACTIN17.4*	5.57	46.15	1275	19	12	7	418	92.8	40.29	−0.057	Cytoplasm	*ACTIN17*
28	g13139	*IbACTIN18*	5.86	62.01	1674	28	18	10	548	85.05	53.27	−0.389	Chloroplast	*ACTIN18*
29	g61314	*IbACTIN19*	7.89	55.85	1533	36	17	10	502	83.92	43.11	0.001	Chloroplast	*ACTIN19*
30	g20278	*IbACTIN20*	5.37	41.79	1152	15	11	8	377	84.62	35.09	−0.19	Cytoskeleton	*ACTIN20*

CDS, coding sequence; MW, molecular weight; pI, isoelectric point; Ser, serine; Thr, threonine; Tyr, tyrosine.

## Data Availability

The data presented in this study are available upon request from the corresponding author.

## Supplementary Materials

The supporting information can be downloaded at: https://www.mdpi.com/article/10.3390/ijms241310930/s1.
