# Supplementary material for: Genome-Wide Identification and Expression Analysis of ACTIN Family Genes in the Sweet Potato and Its Two Diploid Relatives
Source: Int J Mol Sci. 2023 Jun 30;24(13):10930. doi: 10.3390/ijms241310930 (PMC10341734; doi:10.3390/ijms241310930)
Supplement: Supplementary file 1 [file ijms-24-10930-s001.zip › ijms-2391849-supplementary.pdf]

Tabel S1. Identification of ACTIN family genes in *I. batatas, I. trifida,* and *I. triloba*

Arabidopsis	**Homologous gene in *I.batatas/I. trifida/ I.triloba***	**Gene ID**	**Gene name**	**Chromosome localization**
Group I: *AT1G18450.1/AtACTIN2 AT1G73910.3/AtACTIN4*	*I. batatas*	*g5780*	*IbACTIN2.1*	*LG2:10634614-10643350*
		g52926	*IbACTIN2.2*	*LG13:12873733-12882536*
	I. trifida	*itb04g21620.t2*	*ItbACTIN2.1*	*Chr04:26720295-26730623*
		itb04g21620.t3	*ItbACTIN2.2*	*Chr04:26720295-26730623*
		itb04g21620.t1	*ItbACTIN2.3*	*Chr04:26720239-26729819*
		itb02g18870.t1	*ItbACTIN2.4*	*Chr02:15463843-15473934*
	I. triloba	*itf04g22230.t1*	*ItfACTIN2.1*	*Chr04:23942908-23952028*
		itf02g01360.t1	*ItfACTIN2.2*	*Chr02:2012283-2020410*
Group II: *AT3G60830.1/AtACTIN16*	*I. batatas*	*g42892*	*IbACTIN16.1*	*LG11:10263499-10273033*
		g63730	*IbACTIN16.2*	*Chr02:28013749 28019280*
	I. trifida	*itf06g06170.t1*	*ItfACTIN16*	*Chr06:8568303-8573294*
	I. triloba	*itb06g06830.t1*	*ItbACTIN16*	*Chr06:9528314-9533861*
Group III: *AT1G49240.1/AtCTIN3 AT2G42090.1/AtACTIN6 AT2G42100.1/AtACTIN7 AT2G42170.1/AtACTIN8 AT3G18780.2/AtACTIN11 AT5G09810.1/AtACTIN17*	*I. batatas*	*g52274*	*IbACTIN6*	*LG13:8143877-8147254*
		g51848	*IbACTIN7*	*LG13:4939481-4942036*
		g46627	*IbACTIN17.1*	*LG11:38636327-38646881*
		g60046	*IbACTIN17.2*	*LG15:1033740-1036720*
		g27691	*IbACTIN17.3*	*LG7:18085208-18088091*
		g723	*IbACTIN17.4*	*LG1:4228622-4231353*
	I. trifida	*itf02g19390.t1*	*ItfACTIN3*	*Chr02:15433758-15434057*
		itf05g21320.t1	*ItfACTIN17.1*	*Chr05:22330425-22333096*
		itf05g21330.t1	*ItfACTIN17.2*	*Chr05:22338792-22341577*
		itf01g02100.t1	*ItfACTIN17.3*	*Chr01:1256415-1261187*
		itf06g25410.t1	*ItfACTIN17.4*	*Chr06:25189874-25192861*
		itf06g25410.t2	*ItfACTIN17.5*	*Chr06:25189914-25192861*
		itf00g14370.t1	*ItfACTIN17.6*	*Chr00:24154613-24154924*
	I. triloba	*itb05g21980.t1*	*ItbACTIN17.1*	*Chr05:27559059-27561875*
		itb03g12460.t2	*ItbACTIN17.2*	*Chr03:11719074-11721881*
		itb03g12460.t5	*ItbACTIN17.5*	*Chr03:11718987-11722082*
		itb03g12460.t4	*ItbACTIN17.3*	*Chr03:11718969-11721884*
		itb03g12460.t3	*ItbACTIN17.4*	*Chr03:11718987-11722082*
		itb03g12460.t1	*ItbACTIN17.6*	*Chr03:11718969-11722082*
		itb01g02250.t1	*ItbACTIN17.7*	*Chr01:1454234-1458355*
		itb06g24200.t1	*ItbACTIN17.8*	*Chr06:25861466-25864536*

Group IV：<br>*AT2G37620.1/AtACTIN5*<br>*AT3G12110.1/AtACTIN9*<br>*AT3G46520.1/AtACTIN14*<br>*AT3G53750.1/AtACTIN15*<br>*AT5G59370.1/AtACTIN20*	*I. batatas*	*g4902*	*IbACTIN5.1*	*LG2:4782667-4785108*
		g40273	*IbACTIN5.2*	*LG10:15487139-15490779*
		g9979	*IbACTIN5.3*	*LG3:3786950-3788680*
		g18180	*IbACTIN5.4*	*LG5:11043057-11044971*
		g24983	*IbACTIN5.5*	*LG6:29890926-29893324*
		g43021	*IbACTIN5.6*	*LG11:11180492-11182509*
		g3675	*IbACTIN5.7*	*LG1 :26414931-26418418*
		g24981	*IbACTIN5.8*	*LG6:29880896-29885825*
		g25621	*IbACTIN9*	*LG7:2170022-2172796*
		g58376	*IbACTIN15*	*LG14:21779935-21783209*
		g20278	*IbACTIN20*	*LG5:26886916-26889430*
	I. trifida	*itf04g29720.t1*	*ItfACTIN5.1*	*Chr04:29194146-29196490*
		itf08g17480.t1	*ItfACTIN5.2*	*Chr08:17604992-17608424*
		itf08g17480.t2	*ItfACTIN5.3*	*Chr08:17605115-17608424*
		itf14g15680.t1	*ItfACTIN5.4*	*Chr14:16240499-16242679*
		itf01g24130.t1	*ItfACTIN5.5*	*Chr01:24440038-24442798*
		itf05g04190.t1	*ItfACTIN5.6*	*Chr05:3931060-3934026*
		itf05g04190.t2	*ItfACTIN5.7*	*Chr05:3931060-3934175*
		itf15g02700.t2	*ItfACTIN5.8*	*Chr15:1616320-1618673*
		itf15g02700.t1	*ItfACTIN5.9*	*Chr15:1616287-1618760*
		itf15g02720.t1	*ItfACTIN5.10*	*Chr15:1623970-1626886*
		itf03g15730.t1	*ItfACTIN9*	*Chr03:12818363-12821281*
		itf09g12320.t1	*ItfACTIN15*	*Chr09:7282048-7285244*
		itf02g19380.t1	*ItfACTIN20.1*	*Chr02:15433184-15433719*
		itf02g02770.t1	*ItfACTIN20.2*	*Chr02:3821119-3821661*
		itf12g22550.t1	*ItfACTIN20.3*	*Chr12:21077812-21079346*
	I. triloba	*itb04g29160.t2*	*ItbACTIN5.1*	*Chr04:32639044-32641007*
		itb04g29160.t1	*ItbACTIN5.2*	*Chr04:32639044-32641481*
		itb08g13090.t1	*ItbACTIN5.3*	*Chr08:13629731-13633420*
		itb14g17260.t1	*ItbACTIN5.4*	*Chr14:20536211-20538452*
		itb15g02990.t1	*ItbACTIN5.5*	*Chr15:1896361-1897203*
		itb15g02940.t1	*ItbACTIN5.6*	*Chr15:1873904-1874327*
		itb01g23970.t1	*ItbACTIN5.7*	*Chr01:29780602-29783416*
		itb15g02970.t1	*ItbACTIN5.8*	*Chr15:1888710-1889133*
		itb05g03600.t3	*ItbACTIN5.9*	*Chr05:3092648-3095006*
		itb05g03600.t1	*ItbACTIN5.10*	*Chr05:3092648-3095887*
		itb05g03600.t2	*ItbACTIN5.11*	*Chr05:3092648-3095887*
		itb15g03010.t2	*ItbACTIN5.12*	*Chr15:1903507-1905960*

		itb15g03010.t1	*ItbACTIN5.13*	*Chr15:1903507-1905960*
		itb15g03030.t2	*ItbACTIN5.14*	*Chr15:1911558-1914177*
		itb15g03030.t1	*ItbACTIN5.15*	*Chr15:1911499-1914448*
		itb09g20170.t1	*ItbACTIN5.16*	*Chr09:16859717-16862484*
		itb09g20270.t1	*ItbACTIN5.17*	*Chr09:17032924-17036551*
		itb03g16410.t1	*ItbACTIN9*	*Chr03:15383270-15386152*
		itb09g13330.t1	*ItbACTIN15*	*Chr09:8683943-8687216*
		itb02g24040.t1	*ItbACTIN20.1*	*Chr02:24367716-24368489*
		itb12g22900.t1	*ItbACTIN20.2*	*Chr12:24814397-24817275*
Group V: *AT1G13180.1/AtACTIN1 AT3G12380.2/AtACTIN10 AT3G27000.1/AtACTIN12 AT3G33520.1/AtACTIN13 AT5G56180.1/AtACTIN19*	*I. batatas*	*g40378*	*IbACTIN1*	*LG10:16133537-16136575*
		g2351	*IbACTIN10.1*	*LG1:16187825-16194236*
		g2496	*IbACTIN10.2*	*LG1:17381916-17388417*
		g64096	*IbACTIN12.1*	*LG15:30654675-30660529*
		g48567	*IbACTIN12.2*	*LG12:12507463-12510529*
		g18621	*IbACTIN12.3*	*LG5:14393760-14396471*
		g26691	*IbACTIN13*	*LG7:10274134-10278725*
		g61314	*IbACTIN19*	*LG15:9374643-9379415*
	I. trifida	*itf08g11490.t1*	*ItfACTIN1*	*Chr08:10142923-10149460*
		itf05g10940.t1	*ItfACTIN10*	*Chr05:13043140-13049666*
		itf06g08850.t1	*ItfACTIN12.1*	*Chr06:11115639-11119977*
		itf06g08850.t2	*ItfACTIN12.2*	*Chr06:11115639-11118311*
		itf06g08850.t3	*ItfACTIN12.3*	*Chr06:11115654-11118022*
		itf03g23030.t1	*ItfACTIN13.1*	*Chr03:18535286-18540780*
		itf03g23030.t3	*ItfACTIN13.2*	*Chr03:18535287-18540780*
		itf03g23030.t2	*ItfACTIN13.3*	*Chr03:18536189-18540780*
		itf06g15430.t1	*ItfACTIN19.1*	*Chr06:18603669-18608687*
		itf06g15430.t2	*ItfACTIN19.2*	*Chr06:18603669-18608687*
	I. triloba	*itb08g10310.t1*	*ItbACTIN1*	*Chr08:9730655-9737268*
		itb05g11200.t1	*ItbACTIN10*	*Chr05:17179310-17185919*
		itb06g06230.t1	*ItbACTIN12*	*Chr06:8901725-8906191*
		itb03g28750.t1	*ItbACTIN13.1*	*Chr03:29447310-29452052*
		itb03g28750.t3	*ItbACTIN13.2*	*Chr03:29447310-29452052*
		itb03g28750.t2	*ItbACTIN13.3*	*Chr03:29447660-29452051*
		itb06g13650.t1	*ItbACTIN19.1*	*Chr06:18355463-18360340*
		itb06g13650.t2	*ItbACTIN19.2*	*Chr06:18355465-18360337*
		itb06g13650.t3	*ItbACTIN19.3*	*Chr06:18355469-18358337*
Group VI: *AT5G43500.1/AtACTIN18*	*I. batatas*	*g13139*	*IbACTIN18*	*LG4:2514467-2519966*
	I. trifida	*itf13g20930.t1*	*ItfACTIN18.1*	*Chr13:21581222-21586969*

		itf13g20930.t3	*ItfACTIN18.2*	*Chr13:21581222-21586172*
		itf13g20930.t2	*ItfACTIN18.3*	*Chr13:21581222-21586969*
		itf13g20930.t4	*ItfACTIN18.4*	*Chr13:21582313-21586969*
	I. triloba	*itb13g24490.t1*	*ItbACTIN18*	*Chr13:30154071-30162839*

Tabel S2. The Responses of *ItfACTINs* and *ItbACTINs* to hormones.

ID	Groups	ABA	IAA	GA3	BAP
ItfACTIN2.1	GroupI	induced	repressed	induced	induced
ItfACTIN2.2		induced	repressed	induced	induced
ItfACTIN16	GroupII	induced	repressed	induced	induced
ItfACTIN3	GroupIII	-	-	-	-
ItfACTIN17.1		induced	repressed	induced	induced
ItfACTIN17.2		induced	repressed	induced	induced
ItfACTIN17.3		repressed	induced	induced	induced
ItfACTIN17.4		repressed	repressed	repressed	repressed
ItfACTIN17.5		repressed	repressed	repressed	repressed
ItfACTIN17.6		-	-	-	-
ItfACTIN20.1	GroupIV	repressed	repressed	induced	repressed
ItfACTIN20.2		induced	repressed	induced	induced
ItfACTIN20.3		induced	repressed	induced	induced
ItfACTIN9		induced	repressed	induced	induced
ItfACTIN15		induced	repressed	induced	induced
ItfACTIN5.1		induced	repressed	repressed	repressed
ItfACTIN5.2		induced	repressed	induced	induced
ItfACTIN5.3		repressed	induced	repressed	repressed
ItfACTIN5.4		induced	repressed	induced	induced
ItfACTIN5.5		induced	induced	induced	induced
ItfACTIN5.6		induced	repressed	repressed	repressed
ItfACTIN5.7		repressed	repressed	induced	induced
ItfACTIN5.8		repressed	induced	induced	repressed
ItfACTIN5.9		induced	repressed	induced	induced
ItfACTIN5.10		induced	repressed	induced	induced
ItfACTIN12.1	GroupV	induced	induced	induced	induced
ItfACTIN12.2		induced	induced	induced	repressed
ItfACTIN12.3		induced	repressed	induced	induced
ItfACTIN1		induced	repressed	induced	induced
ItfACTIN13.1		repressed	repressed	repressed	induced
ItfACTIN13.2		induced	induced	induced	induced
ItfACTIN13.3		induced	induced	induced	repressed
ItfACTIN10		repressed	repressed	induced	repressed
ItfACTIN19.1		repressed	repressed	induced	repressed
ItfACTIN19.2		repressed	repressed	induced	repressed
ItfACTIN18.1	GroupVI	repressed	repressed	induced	induced
ItfACTIN18.2		induced	induced	induced	induced
ItfACTIN18.3		induced	induced	repressed	induced
ItfACTIN18.4		repressed	repressed	induced	repressed
ItbACTIN2.1	GroupI	induced	induced	induced	induced

ItbACTIN2.2	GroupI	repressed	repressed	repressed	repressed
ItbACTIN2.3		induced	repressed	induced	induced
ItbACTIN2.4		induced	induced	induced	induced
ItbACTIN16	GroupII	repressed	repressed	induced	induced
ItbACTIN17.1	GroupIII	induced	repressed	induced	repressed
ItbACTIN17.2		repressed	repressed	repressed	repressed
ItbACTIN17.3		repressed	induced	repressed	induced
ItbACTIN17.4		repressed	induced	induced	induced
ItbACTIN17.5		induced	induced	induced	induced
ItbACTIN17.6		repressed	repressed	induced	induced
ItbACTIN17.7		induced	repressed	repressed	repressed
ItbACTIN17.8		induced	repressed	repressed	repressed
ItbACTIN20.1	GroupIV	repressed	induced	induced	induced
ItbACTIN20.2		repressed	induced	induced	induced
ItbACTIN9		induced	repressed	induced	induced
ItbACTIN15		induced	repressed	induced	repressed
ItbACTIN5.1		induced	induced	repressed	repressed
ItbACTIN5.2		repressed	induced	repressed	induced
ItbACTIN5.3		repressed	repressed	induced	repressed
ItbACTIN5.4		repressed	repressed	induced	induced
ItbACTIN5.5		induced	repressed	repressed	induced
ItbACTIN5.6		repressed	induced	induced	induced
ItbACTIN5.7		repressed	repressed	induced	induced
ItbACTIN5.8		-	-	-	-
ItbACTIN5.9		induced	induced	repressed	induced
ItbACTIN5.10		repressed	induced	repressed	induced
ItbACTIN5.11		repressed	repressed	induced	induced
ItbACTIN5.12		-	-	-	-
ItbACTIN5.13		-	-	-	-
ItbACTIN5.14		induced	repressed	repressed	induced
ItbACTIN5.15		induced	induced	induced	repressed
ItbACTIN5.16		-	-	-	-
ItbACTIN5.17		-	-	-	-
ItbACTIN12	GroupV	induced	repressed	induced	induced
ItbACTIN1		induced	repressed	induced	induced
ItbACTIN13.1		induced	repressed	repressed	induced
ItbACTIN13.2		induced	induced	induced	induced
ItbACTIN13.3		repressed	repressed	induced	repressed
ItbACTIN10		induced	repressed	induced	induced
ItbACTIN19.1		repressed	induced	repressed	induced
ItbACTIN19.2		repressed	repressed	induced	repressed
ItbACTIN19.3		repressed	repressed	repressed	repressed
ItbACTIN18	GroupVI	induced	repressed	induced	induced

- indicates not found

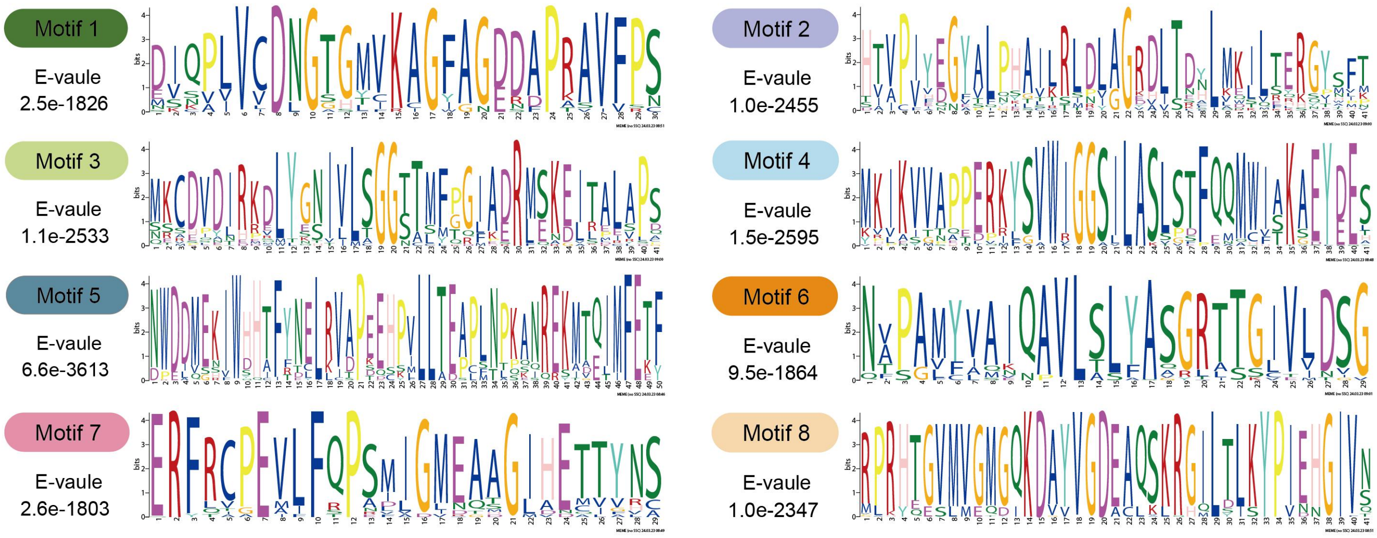

Figure S1. Conserved motifs analysis of IbACTINs in *I. batatas*.
